# A Special Extract of *Bacopa monnieri* (CDRI-08) Restores Learning and Memory by Upregulating Expression of the NMDA Receptor Subunit GluN2B in the Brain of Scopolamine-Induced Amnesic Mice

**DOI:** 10.1155/2015/254303

**Published:** 2015-08-27

**Authors:** Rakesh Rai, Hemant K. Singh, S. Prasad

**Affiliations:** ^1^Biochemistry and Molecular Biology Laboratory, Brain Research Centre, Department of Zoology, Banaras Hindu University, Varanasi, Uttar Pradesh 221 005, India; ^2^Lumen Research Foundation, Ashok Nagar, Chennai 600083, India

## Abstract

In the present communication, we have investigated effects of the CDRI-08, a well characterized extract of* Bacopa monnieri*, on expression of the GluN2B subunit of NMDAR in various brain regions of the scopolamine-induced amnesic mice. Our behavioral data reveal that scopolamine-treated amnesic mice exhibit significant decline in the spatial memory compared to the normal control mice. Our RT-PCR and immunoblotting data revealed that the scopolamine treatment resulted in a significant downregulation of the NMDAR GluN2B subunit expression in prefrontal cortex and hippocampus. Our enzyme assay data revealed that scopolamine caused a significant increase in the acetylcholinesterase activity in both the brain regions. Further, oral administration of the CDRI-08 to scopolamine-treated amnesic mice restored the spatial memory which was found to be associated with significant upregulation of the GluN2B subunit expression and decline in the acetylcholinesterase activity in prefrontal cortex as well as hippocampus towards their levels in the normal control mice. Our study provides the evidence for the mechanism underlying role of the* Bacopa monnieri* extract (CDRI-08) in restoring spatial memory in amnesic mice, which may have therapeutic implications.

## 1. Introduction 

Amnesia is characterized by deficit in memory caused by either brain damage, neurological disorders, psychological trauma [1], use of sedative/hypnotic drugs [2], or alcohols [3] due to alterations in the excitatory glutamatergic synaptic strength, which is dependent on the activation of the ionotropic glutamate receptors, AMPA (*α*-amino-3-hydroxy-5-methyl-4-isoxazole propionic acid) or NMDA (N-methyl-D-aspartic acid) receptors (AMPAR or NMDAR), and metabotropic glutamate receptors (mGluRs) [4]. Studies on the NMDAR mutant mice have shown that activity dependent hippocampal CA1 synaptic plasticity is abrogated due to the absence of NMDAR [5]. Large body of evidences suggests that expression of calcium/calmodulin-dependent protein kinase II (CaMKII), brain-derived neurotrophic factor (BDNF), and calcineurin is differentially altered in the hippocampus, basolateral amygdala (BLA), and medial prefrontal cortex (mPFC) stress-induced amnesia [6]. However, information on alterations in the expression of NMDA receptor or its particular subunit in the drug-induced amnesic animal model is less studied.

N-Methyl-D-aspartate (NMDA) receptors, a heterotetrameric structure, consist of two GluN1 subunits and two additional GluN2 or GluN3 subunits which together confer the functionality to the receptor. Each subunit possesses the N-terminal domain containing binding sites for allosteric regulators such as Zn^2+^, the agonist binding domain for glycine/D-serine (GluN1) and glutamate (GluN2) where competitive antagonists bind [4]. GluN1 has eight splice variants whereas GluN2 subunit consists of four splice variants (NR2A-D). GluN1 subunit is an obligatory component of the NMDA receptors whereas variation takes place at the level of GluN2 subunit types. The GluN2 subunit type present in NMDA receptor complex critically determines its biophysical, pharmacological, and physiological properties including sensitivity to Zn^2+^, H^+^ and polyamines, single channel conductance, and interactions with intracellular signaling molecules [7, 8]. GluN2A and GluN2B are associated with higher brain functions [9, 10] and they are predominantly expressed in hippocampus and cortex [4, 11]. Recent evidence indicates that NMDA receptor activity is correlated with learning, memory, and cognition by modulating dendritic spine density, synaptic plasticity, and synaptic strength [12, 13]. Also, GluN2A and GluN2B subunits of NMDARs are implicated in the development of LTP in the hippocampus-dependent spatial and fear memory, and they have been correlated with overexpression of GluN2B subunit in order to enhance the above memory forms in adult mice [14]. Recent studies have implicated possible role of NMDA receptors in various neurological disorders like epilepsy, Alzheimer disease, and Huntington chorea and mild cognitive impairment (MCI) [7, 15]. Pharmacological and knockout studies have demonstrated that mice lacking GluN2A/B subunits exhibit impaired LTP and thereby deficient spatial memory [16]. GluN2B subunit of the NMDA receptor has been shown to be associated with altered synaptic plasticity in Parkinson's disease [17, 18]. Therefore, investigation on alterations in the expression of the GluN2B in the amnesic mice may provide an important support to involvement of NMDAR in learning and memory and which may also serve as a measure to evaluate the molecular mechanism of effects of herbal neuromodulator drugs such as* Bacopa* extract. Therefore, we have analyzed alterations in the expression of the NMDA receptor subunit GluN2B in prefrontal cortex and hippocampus of the scopolamine-treated amnesic mice [19, 20] and investigated the potential effects of* Bacopa monnieri* extract on its alterations during experimental amnesia.

*Bacopa monnieri* is a traditional herbal plant and its extract has been used in Indian medicine system since ancient period as a nerve tonic for the treatment of varieties of neurological diseases and memory related disorders [21, 22]. CDRI-08 is a well characterized extract of* Bacopa monnieri* and it contains several active phytochemical constituents such as bacosides A and B, alkaloids, and saponins. Bacosides A and B [23–25] have been used in the treatment of neurological disorders like insomnia, depression, anxiety, psychosis, and stress [26–28]. Several studies have demonstrated their (bacosides A and B) antiamnesic, antiepileptic, neuroprotective, and memory enhancing effects [21, 29–31]. The number of studies carried out on the mechanisms of antiamnesic action of* Bacopa monnieri* extract indicates that it improves the working memory and cognition in elderly human subjects by reducing plasma acetylcholinesterase activity (AChE) [32]. Evidence from studies on the effects of* Bacopa monnieri* extract on the scopolamine-induced amnesic mice suggests that it reverses the state of amnesia by significantly improving calmodulin level and by partially attenuating the protein kinase C and pCREB activities [33]. However, literatures on the effects of the* Bacopa monnieri* extract particularly the CDRI-8 on alterations in the AChE activity and its possible correlation with expression of the NMDA receptor subunit GluN2B especially in various brain regions in animal models of experimental amnesia are lacking.

To investigate above, amnesic mice model was developed by intraperitoneal injection of scopolamine (2 mg/kg BW), examined the mice for their spatial memory impairment by eight-arm radial maze test and studied its possible correlations with altered expression of NMDAR GluN2B subunit in the prefrontal cortex and hippocampus using Western blotting and semiquantitative RT-PCR techniques. Further, to examine the neuroprotective effects of CDRI-08 via NMDA receptor, the amnesic mice were treated with standardized dose of CDRI-08 compared with vehicle-treated normal and CDRI-08-treated control mice, separately. Since, scopolamine is a known nonselective muscarinic acetylcholine receptor antagonist, which in turn blocks the effects of acetylcholine, impairs LTP, and induces amnesia in mammals [34], and it has been widely used to induce amnesia in animal models, we also examined alterations in the activity of acetylcholinesterase to validate the scopolamine's anticholinergic effects which might lead to accumulation of the synaptic acetylcholine content and thereby increase in the activity of acetylcholinesterase. Here, we report that scopolamine-induced amnesia is associated with decline in the expression of NMDA receptor GluN2B subunit in both the brain regions and the CDRI-08 reverses the memory loss by upregulating its expression close to the vehicle-treated normal control mice.

## 2. Materials and Methods

### 2.1. Animals

Male Swiss strain albino mice of 20 ± 2 weeks were used throughout the experiment and they were maintained in the animal house at 24 ± 2°C with 12 hr light/dark cycle and fed with standard mice feed and water* ad libitum*. Mice were used as per norms set by animal ethical clearance committee of Banaras Hindu University.

### 2.2. Chemicals, Drugs, and Antibodies

Chemicals used in experiments were of molecular biology grades and were purchased from Sigma, USA, or Merck, India. The specialized extract of* Bacopa monnieri* extract containing bacosides A and B (CDRI-08) was obtained from Mr. S. Selvam, Lumen Research Foundation, Chennai, India, as a gift and was suspended in Tween 80 (0.5% v/v). Scopolamine was purchased from Sigma-Aldrich, New Delhi, and was dissolved in normal saline. Drugs solutions were freshly prepared at the time of use. Anti-GluN2B primary antibody was obtained from Antibodies Incorporated (Neuromab, UC Davis, USA) and HRP-conjugated secondary antibody raised in goat against anti-mouse primary antibodies was purchased from Genie, Bangalore, India.

### 2.3. Animal Groups and Drug Treatment Schedule for Acquisition Study

Mice were divided into four groups as shown in Table 1. Each group comprised of 7 mice.* Group I* (control): mice in this group received oral administration of 0.5% Tween 80 in normal saline medium followed by intraperitoneal injection of normal saline and were subjected to radial arm maze test daily for three weeks;* Group II* (*Bacopa monnieri* extract (CDRI-08) treated): mice in this group were treated daily for one week period as in the control group and subjected to radial arm maze tests. They were further treated by oral administration of CDRI-8 (200 mg/Kg BW) in 0.5% Tween 80 diluted with normal saline as a medium for two weeks and subjected to radial arm maze test daily two hours after the treatment. This group was prepared to study the effects of CDRI-08 on the normal control mice;* Group III* (scopolamine-treated): mice in this group were treated, with normal saline and 0.5% Tween 80, as in the control group and subjected to radial arm maze test daily for two weeks. Thereafter, these mice were treated with intraperitoneal injection of scopolamine (2 mg/Kg BW in normal saline) followed by oral treatment of 0.5% Tween 80 diluted with normal saline for the third week. These mice were subjected to radial arm maze test daily two hrs after the treatments;* Group IV* (scopolamine and CDRI-08-treated): mice in this group were treated orally with 0.5% Tween 80 and with intraperitoneal injection of scopolamine as in Group III daily for one week. Thereafter, these mice were treated orally with CDRI-08 (200 mg/Kg BW) daily as in Group II for two weeks and each mouse in the group was subjected to radial arm maze test two hrs after treatments. The time gap of two hrs between drug treatment and the radial arm maze was chosen to avoid any possible alterations in the motor activities of mice compared to 30–90 min time gaps reported in literatures wherein researchers have used 0.5–1.0 mg scopolamine/Kg BW. Thus mice belonging to all four groups were subjected to three weeks of behavioral test on the eight-arm radial maze paradigm equally.

The final tests were performed on the 22nd day as has been described in Section 3.

## 3. Methodology

### 3.1. Eight-Arm Radial Maze Test

Each behavioral test session for studying acquisition and memory after the vehicle or drug treatment was conducted in standard eight-arm radial maze (RAM) equipment consisting of a central platform of a 25 cm diameter with eight arms of 70 cm (length) × 10 cm (width) × 15 cm (height) each, radiating at equal angle from the central platform. The maze was placed at a fixed position to reduce the variability of each test. In the present study, baited and unbaited arms were fixed throughout the tests. The 1st, 3rd, 5th, and 7th arms were baited (with food) while the 2nd, 4th, 6th, and 8th arms were unbaited (without food). At the very beginning of each test session, each mouse was placed in the central platform of the equipment at the position facing towards the 1st arm. Food-deprived mice were expected to seek specific arms with rewards and subsequently register and retain the memory of each entered arm where food was present. Each mouse was allowed to freely explore and consume food rewards for 3 minutes or until all food rewards of the four baited arms were eaten, which ever occurred first. An entry was recorded every time when the mice placed all four paws into the initial part of the arm. The maze was then thoroughly cleaned with 70% alcohol prior to the next test session in order to minimize the effect of residual odors of food from previous tests [35]. The first entry into never-baited arms was scored as a reference memory error (RME), reentry into arms where the food reward had already been eaten was scored as a working memory error (WME), and reentry into unbaited arm is considered reference-working memory errors (RWE) [36, 37].

### 3.2. Brain Tissue Harvesting and Processing

After the radial arm maze tests were completed on every individual mouse of each experimental set separately, mice of each group were sacrificed by cervical dislocation. Whole brain was immediately removed and washed with ice cold normal saline. Prefrontal cortex and hippocampus were dissected out on ice and blotted dry quickly within the folds of blotting paper and pooled and used directly for the neurochemical studies or stored frozen at −70°C.

### 3.3. Assay of Acetylcholinesterase (AChE) Activity

AChE activity was measured using modified Ellman's colorimetric method [38, 39]. Briefly, hippocampal and prefrontal cortical tissue of brain were quickly homogenized in 0.1 M phosphate buffer, pH 7.4, separately. The acetylcholinesterase activity was measured by adding an artificial substrate analog of acetylcholine, acetylthiocholine (ATC) for every two min. Thiocholine released because of the cleavage of ATC by AChE is allowed to react with the –SH group of the reagent 5, 5′-dithiobis-(2-nitrobenzoic acid) (DTNB), which is reduced to thionitrobenzoic acid, a yellow colored anion with an absorbance maxima at 412 nm. The molar extinction coefficient of the thionitrobenzoic acid was taken as 1.36 × 10^4^/M/cm. The concentration of thionitrobenzoic acid was determined using a UV-Vis spectrophotometer and the AChE activity was calculated using the formula: (*R* = 5.74 × 10^4^ XA)/CO, where *R* = rate in moles of substrate hydrolyzed/min/gm wet wt of tissue; *A* = change in absorbance/min; and CO = original concentration of the tissue (mg/mL).

### 3.4. Prefrontal Cortex and Hippocampal Lysate Preparation

The prefrontal or hippocampus tissue was homogenized in TEEN buffer (50 mM Tris-HCl, pH 7.4, 1 mM EDTA, 1 mM EGTA, 150 mM NaCl) supplemented with 2 mM PMSF and 1 *μ*g/mL protease inhibitor cocktail. Thereafter, the homogenate was centrifuged at 5000 ×g. The resulting supernatant was collected and aliquoted in small fractions. The total protein content in the lysate was estimated by Bradford method [40]. Aliquots were directly used for further experiment or stored at −70°C.

### 3.5. Western Blotting

The prefrontal or hippocampal lysate was boiled at 100°C for 5 min in SDS containing sample loading buffer (10 mM Tris-HCl pH 6.8, 0.2% *β*-mercaptoethanol, 2% SDS, 20% glycerol) and centrifuged at 10000 ×g at 4°C for 20 min. The supernatant was carefully collected. 50 *μ*g total protein was resolved on 7.5% SDS-polyacrylamide gel as described earlier [41]. Thereafter, proteins from the gel were immobilized onto polyvinylidene difluoride (PVDF) membrane by wet transfer method. To ensure the transfer of proteins, the membrane was stained with Ponceau-S. The PVDF membrane was washed in 1x phosphate buffer saline (PBS) and was blocked with 5% nonfat milk powder dissolved PBS for 4 h at RT. Thereafter, the membrane was incubated with anti-GluN2B antibody (1 : 2000) overnight and washed for 5 min in PBST (PBS containing 10 mM Tris-HCl, pH 7.0). The blots were also processed with rabbit monoclonal anti-*β*-actin antibody (1 : 25,000, Sigma-Aldrich, USA) in parallel in order to examine the level of *β*-actin as internal control. Thereafter, membranes were incubated with goat anti-mouse HRP-conjugated secondary antibody (1 : 2500 in PBS containing 5% nonfat milk) for 4 h and then washed with PBST at RT. The specific protein-antibody complex on the membrane was detected by enhanced chemiluminescence (ECL) method following the manufacturer's protocols. Resulting signals on the X-ray film were densitometrically scanned individually and quantified by computer-assisted densitometry (Alpha imager 2200). Scan data of individual proteins were normalized with that of the *β*-actin to obtain relative density value (RDV) for GluN2B.

### 3.6. Isolation of Total RNA

Total RNA from the prefrontal cortex or hippocampus was isolated using TRI reagent (Sigma, USA) following the suppliers' manual. The aqueous phase was collected and mixed with equal volume (v/v) of isopropanol and precipitated at −70°C. The RNA pellet was collected, washed with ice-chilled 70% ethanol, and dissolved in diethylpyrocarbonate- (DEPC-) treated water. Extracted RNA was treated with DNase-I (DNA-free, Ambion) according to the manufacturer's guidelines to remove any DNA contamination. RNA content was determined by measuring the absorbance at 260 nm using UV-Vis spectrophotometer. Integrity of the RNA samples was checked by 1% formaldehyde agarose gel electrophoresis [41].

### 3.7. Semiquantitative RT-PCR

cDNA from total RNA was synthesized by mixing 2 *μ*g of the DNA-free total RNA and 200 ng random hexamer primers (MBI Fermentas, USA) in 11 *μ*L reaction volume and incubating the whole mix at 70°C for 5 min. Thereafter, 2 *μ*L of 5x reaction buffer, 2 *μ*L of 10 mM dNTP mix, and 20 U of RNase inhibitor (Ribolock, MBI Fermentas, USA) were added, and the volume was made up to 19 *μ*L. The tube was incubated for 5 min at 25°C, and 200 U of M-MuLv reverse transcriptase (RT) (New England Biolabs) was added. Further, the tube was incubated for 10 min at 25°C initially and then at 42°C for 1 h in the Thermal Cycler (G-Storm, UK). The reaction was terminated by heating the reaction mix at 70°C for 10 min followed by its incubation at 4°C. PCR reactions were carried out in a 25 *μ*L reaction mixture containing 2 *μ*L cDNA, 1x Taq polymerase buffer with MgCl_2_, 0.2 mM of each dNTP (MBI Fermentas, USA), 1.0 unit of Taq DNA polymerase (Banglore Genei, India), and 10 pmol of appropriate primers for GluN2B (F-CTGGATTCTGCATTGTGAGC, R-CACGAGGATGACAGCGATG) and *β*-actin (F-ATCGTGGGCCGCTCTAGGCACC, R-CTCTTTGATGTCACGATTTC) in Thermal Cycler (G-Strom, UK) for 28 cycles. Each PCR amplified product was individually mixed with 6x loading dye (30% glycerol, 0.25% bromophenol blue, and 0.25% xylene cyanol) and were resolved separately by 2% agarose gel electrophoresis using a tank buffer 1x TAE buffer (40 mM Tris, 40 mM acetic acid, and 1 mM EDTA) containing ethidium bromide. The DNA bands were visualized in UV transilluminator and images of the gel were captured. The image was densitometrically scanned separately and quantified using Fluorchem software, version 2.0 (Alpha Innotech, USA). Integrated density value (IDV) of the GluN2B specific DNA band was normalized with IDV of the *β*-actin DNA band to obtain relative density value (RDV).

### 3.8. Statistical Analysis

All the neurochemical experiments were repeated at least three times taking a batch of 6-7 mice per experimental group. The RDV data was presented as bar diagram showing mean ± SEM and the data were analyzed by one-way ANOVA between experimental groups followed by post hoc Bonferroni multiple comparison tests using two-tailed *P* values with SPSS-16. The *P* values <0.05 were taken as significant. Performance of mice from each experimental group on the radial arm maze test for the analysis of various memory types during retention test was analyzed by one-way ANOVA followed by Bonferroni tests and learning curves for percent correct memory were analyzed as trial blocks for three trials (one trial block/day).

## 4. Results 

### 4.1. Acquisition Processes Is Not Affected by Tween 80 and Normal Saline

**Figure 1:**
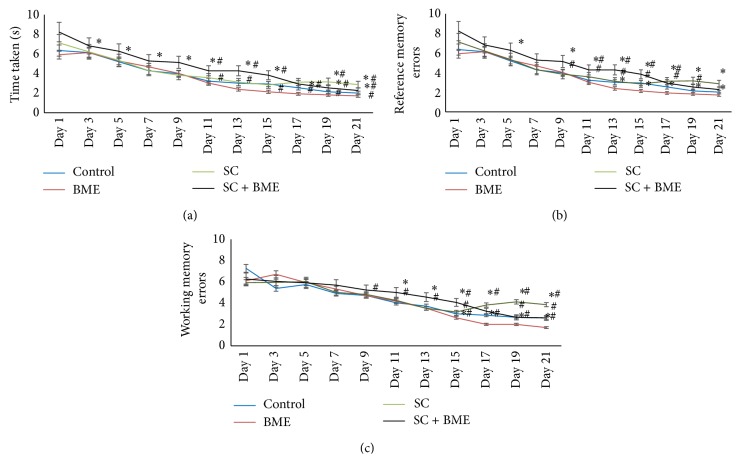
Radial arm maze analysis of acquisition by mice during training period. Mouse of each experimental group was individually trained on the maze for searching food and time spent; reference memory errors, working memory errors, and reference-working memory errors were recorded. Graphs represent average value ± SEM of above parameters during acquisition trials. Data were analyzed by repetitive measures ANOVA followed by Dunnett's post hoc tests. ∗, *P* < 0.05 for mice groups in comparison to control within same day; #, *P* < 0.05 for mice groups on a particular day in comparison to that mice within groups on day 1. (a) Time taken to retrieve hidden food in radial arm maze. (b) and (c) Reference memory errors and working memory errors.

To understand whether Tween 80 or normal saline per se have any impact on the acquisition and memory processes, the control mice were subjected to radial arm maze test.  Figure 1 reveals the results of acquisition and memory during vehicle or the drug treatment period at the level of latency time in general, and reference and working memory at the levels they incorporate errors while entering in the nonbaited and baited arms of the RAM, respectively. As is evident from Figure 1(a), treatment of mice with the drug vehicle, that is, Tween 80 or normal saline for various durations, did not affect the learning and memory processes. The behavioral analysis data reveal that all the mice in control set were able to learn during training period from day 1 in the first week till day 21 in the third week. Tween 80 and normal saline did not affect their abilities of learning and memory at the level of latency time and incorporation of errors.

### 4.2. *Bacopa monnieri* Extract Reverses Learning Defects and Corrects the Scopolamine-Induced Spatial Memory Loss

It is evident from the behavioral data obtained from radial arm maze test as shown in Figures 1(a), 1(b), and 1(c) that scopolamine treatment leads to significant decline in the acquisition and* Bacopa monnieri* extract (CDRI-08) treatment significantly improved it towards that in the normal control mice.

**Figure 2:**
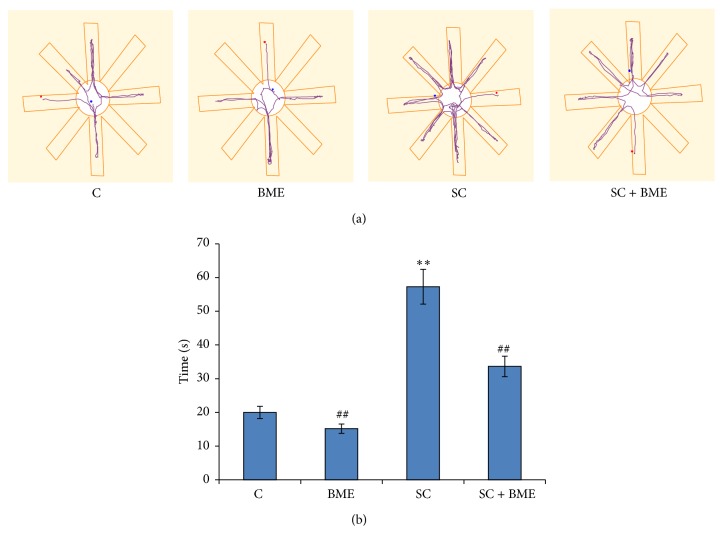
Radial arm maze analysis of spatial memory of mice of control and experimental groups: track record of movement mice in radial arms (a). Bar diagram showing the latency time for retrieving the hidden food (b). Mouse of each group was individually subjected to radial arm maze test and the time taken for retrieving food was recorded. Data represents mean ± SEM. C, vehicle-treated control; BME,* Bacopa monnieri* extract (CDRI-08) treated (200 mg/Kg/BW); SC, scopolamine-treated (2 mg/Kg BW); SC + BME, scopolamine-treated mice treated with CDRI-08. ∗ and #, *P* < 0.05 and ## and ∗∗, *P* < 0.01 were considered significant. ∗, comparison between control and other groups, and #, comparison between SC and other groups.

Figure 2(a) shows track plot report of mice of different experimental groups. It reveals that CDRI-08-treated mice showed fewer errors and were able to track the food more accurately as compared to control group. However, the scopolamine-treated mice experienced problems in locating the hidden food pellet with significantly more errors in above process. CDRI-08-treated mice significantly improved their performance with fewer errors nearer to the normal control. The CDRI-08 treatment to amnesic mice restored the memory for locating food pellets compared to scopolamine-treated mice. Our Any Maze Software Analysis data on the eight-arm radial maze test reveal that scopolamine-treated mice exhibit significant impairment in memory retrieval (amnesia) compared to that in the normal control mice (*P* < 0.01).* Bacopa monnieri* extract CDRI-08 treatment reverses the condition of scopolamine-induced amnesia (*P* < 0.01). It was also observed that mice treated with CDRI-08 alone also showed significant improvement in the memory retention (*P* < 0.01) (Figures 2(a) and 2(b)).

**Figure 3:**
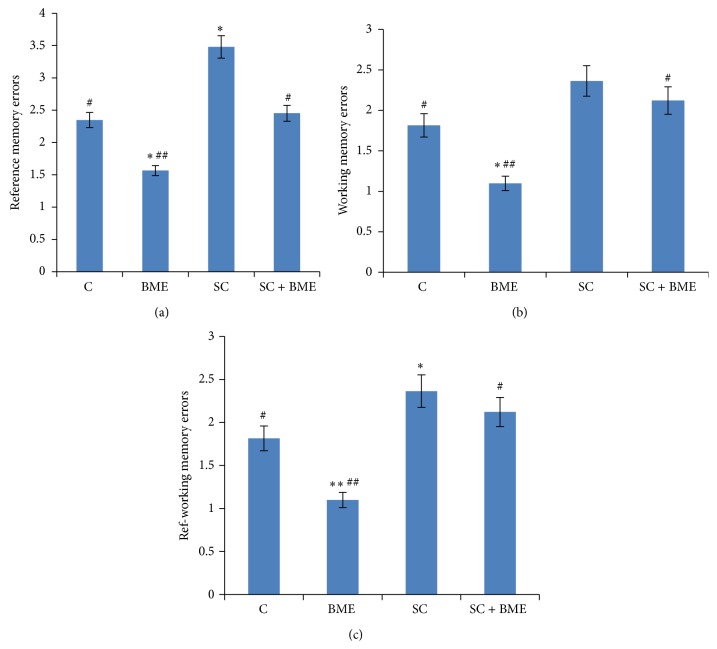
Radial arm maze tests for reference memory error (a), working memory error (b), and reference-working memory error (c). Mouse of each group was individually subjected to radial arm maze test for recording the errors. Data represents mean ± SEM. C, control; BME (*Bacopa monnieri* extract), CDRI-08-treated; SC, scopolamine-treated; SC + BME, scopolamine-treated mice treated with CDRI-08 as in Figure 2. ∗ and #, *P* < 0.05 and ## and ∗∗, *P* < 0.01 were considered significant. ∗, comparison between control and other groups. #, comparison between SC and remaining groups.

Figure 3 reveals alterations in various memory forms such as working memory, reference memory, and reference-working memory on the 22nd day based on producing errors during entries in the baited arms (in which the food reward was kept) or nonbaited arms (where there was no food reward).  Figure 3(a) shows errors in reference memory shown by the mice of different groups. Our observations reveal that the scopolamine-treated amnesic mice commit significantly more errors in retrieving the reference memory as compared to that in the normal control mice (*P* < 0.05). It suggests that these mice lose the ability to remember the exact location of food in respect of the arms which were not baited. Mice of control group treated with CDRI-08 produced significantly fewer reference memory errors in comparison to normal control mice (*P* < 0.01). The scopolamine-treated mice (amnesic) after the treatment with CDRI-08 produced significantly less number of errors while retrieving the reference memory during location of food (*P* < 0.05). Scopolamine was found also to significantly reduce the working memory in mice compared to normal control (*P* < 0.05). CDRI-08 alone was found to improve the working memory significantly compared to its effect on the normal control mice (*P* < 0.05). Scopolamine-treated amnesic mice after the treatment of CDRI-08 showed significantly improved performance on the maze test (*P* < 0.05) (Figure 3(b)).

Figure 3(c) shows alterations in reference-working memory in mice belonging to different experimental groups. Our data suggest that the scopolamine treatment of mice leads to significant decline in the RWM compared to that in the normal control mice. Significant improvement in the reference-working memory (RWM) was observed in amnesic mice after treatment with CDRI-08 (*P* < 0.05). It was observed that the CDRI-08 treatment alone was also able to boost the RWM compared to that in the normal control mice (*P* < 0.01). Thus the quantitative analysis of RAM behavior data reveals that* Bacopa monnieri* extract (CDRI-08) does have positive effects on the normal control mice at above scales of learning and memory and it has altogether neuroprotective effects. Further, the treatment of scopolamine-induced mice with* Bacopa monnieri* extract (CDRI-08) has a precognitive effect on learning and memory lost due to scopolamine-induced amnesia.

### 4.3. Scopolamine Enhances the AChE Activity in Prefrontal Cortex and Hippocampus and* Bacopa monnieri* Extract (CDRI-08) Reverses This Effect

**Figure 4:**
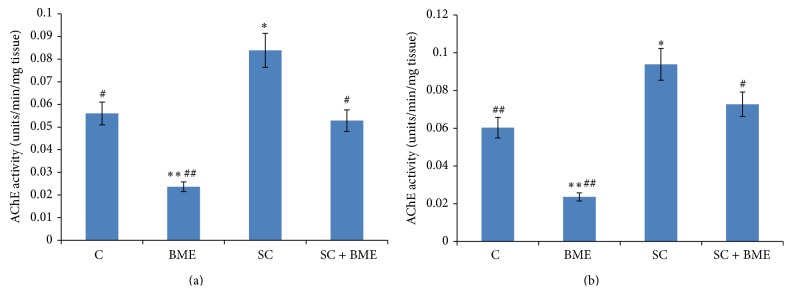
Acetylcholinesterase activity in prefrontal cortex (a) and hippocampus (b). Tissues obtained from 6-7 mice of each group were pooled and AChE activity was assayed. The AChE activity was expressed as unit/min/mg tissue. Data represents mean ± SEM. C, control; BME (*Bacopa monnieri* extract), CDRI-08-treated; SC, scopolamine-treated; SC + BME, scopolamine-treated mice treated with CDRI-08 as in Figure 2. ∗ and #, *P* < 0.05 and ## and ∗∗, *P* < 0.01 were considered significant. ∗, comparison between control and other groups. #, comparison between SC and remaining groups.

Our observations on the assay of AChE activity reveal that its activity is significantly increased in the prefrontal cortex of scopolamine-treated amnesic mice when compared with that in the normal control mice (*P* < 0.05). CDRI-08 treatment to amnesic mice results in significant decline in the AChE activity and the CDRI-08 treatment when given to normal control mice; the AChE activity is significantly reduced (*P* < 0.05) (Figure 4(a)).  Figure 4(b) shows the patterns of AChE activities in the hippocampus of mice of different experimental groups. Scopolamine-treated mice exhibited significant increase in the AChE activity (*P* < 0.05) compared to that in the hippocampus of the normal control mice. CDRI-08 treatment of the amnesic mice significantly decreases the AChE activity toward that in the normal control mice (*P* < 0.05). Also, the CDRI-08 alone significantly decreases the activity in the hippocampus when compared to that in the normal control mice (*P* < 0.01).

### 4.4. Scopolamine Downregulates the Expression of GluN2B in Prefrontal Cortex and Hippocampus and* Bacopa monnieri* Extract (CDRI-08) Recovers It towards Normal

**Figure 5:**
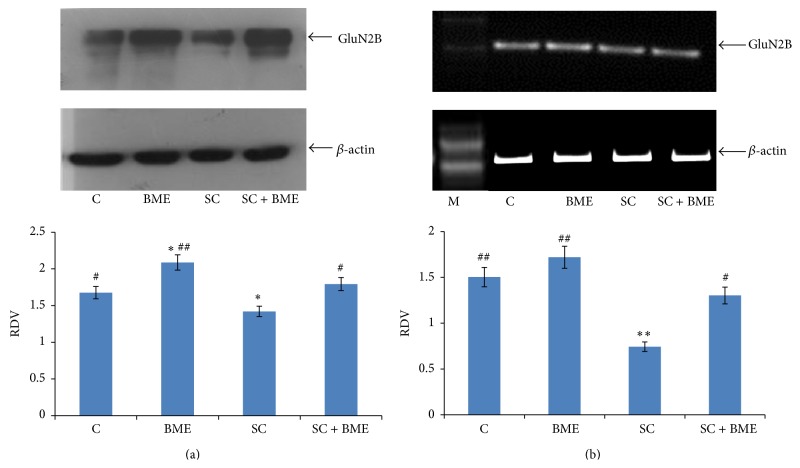
Western blot (a) and semiquantitative RT-PCR (b) analysis of GluN2B expression in prefrontal cortex. Prefrontal cortex from 6-7 mice of each group was pooled; lysates were prepared and detected for presence of GluN2B by ECL. X-ray film was scanned and the data was expressed as relative density value (RDV) by dividing the integrated density value of GluN2B by IDV of the *β*-actin. The data represents mean ± SEM. C, control; BME (*Bacopa monnieri* extract), CDRI-08-treated; SC, scopolamine-treated; SC + BME, scopolamine-treated mice treated with CDRI-08 as in Figure 2. ∗ and #, *P* < 0.05 and ## and ∗∗, *P* < 0.01 were considered significant. ∗, comparison between control and other groups. #, comparison between SC and the remaining groups.

**Figure 6:**
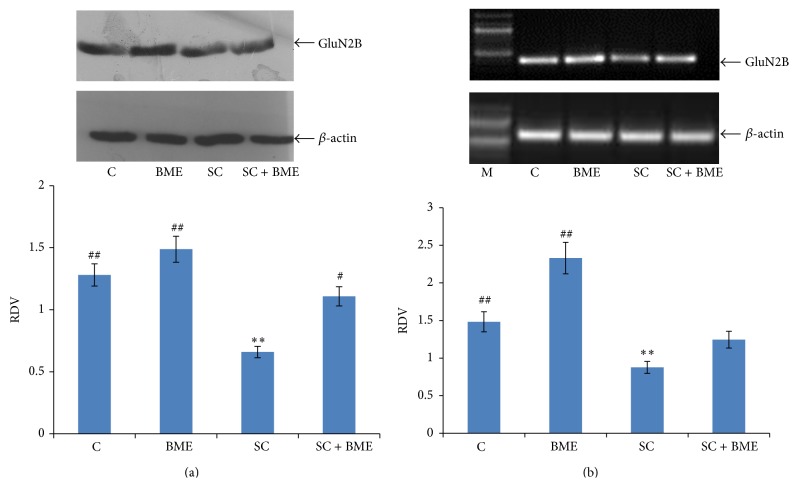
Western blot (a) and semiquantitative RT-PCR (b) analysis of GluN2B expression in hippocampus. Hippocampus from 6-7 mice of each group was pooled; lysates were prepared and detected for presence of GluN2B by ECL. X-ray film was scanned and the data was expressed as relative density value (RDV) by dividing the integrated density value of GluN2B by IDV of the *β*-actin. The data represents mean ± SEM. C, control; BME (*Bacopa monnieri* extract), CDRI-08-treated; SC, scopolamine-treated; SC + BME, scopolamine-treated mice treated with CDRI-08 as in Figure 2. ∗ and #, *P* < 0.05 and ## and ∗∗, *P* < 0.01 were considered significant. ∗, comparison between control and other groups. #, comparison between SC and the remaining groups.

Our immunoblot data reveals that the scopolamine treatment significantly downregulates the level of GluN2B subunit expression in the prefrontal cortex (*P* < 0.05) (Figure 5(a)) and the hippocampus when compared to that in the normal control mice (*P* < 0.05) (Figure 6(a)). CDRI-08 treatment to scopolamine-treated (amnesic mice) significantly upregulates the expression of GluN2B subunit toward that in the normal control mice in both the brain regions. Further, the CDRI-08 alone also is found to significantly upregulate the expression of the GluN2B in the prefrontal cortex as well as hippocampus (*P* < 0.01) (Figures 5(a) and 6(a)). Our semiquantitative RT-PCR data on the expression of GluN2B transcript largely resembles with those of our immunoblot data for both the prefrontal cortex and the hippocampus (Figures 5(b) and 6(b)).

## 5. Discussion

We have studied whether expression of GluN2B in the prefrontal cortex and hippocampus of amnesic mouse model is altered in order to understand the mechanisms of scopolamine-induced amnesia involving one of the ionotropic glutamate receptors such as NMDA receptors and effects of* Bacopa monnieri* extract CDRI-08 during recovery of memory loss in mice. Also, we have investigated the effects of CDRI-08 on the expression on this subunit of the NMDA receptor as it plays important role in synaptic plasticity which underlies learning and memory. Since CDRI-08 has been in use as memory booster drug, we checked its role in the recovery of impaired learning and memory due to amnesia using eight-arm radial maze paradigm. We have used scopolamine for inducing amnesia in mice in the current study. As the scopolamine is a muscarinic acetylcholine receptor antagonist, it may lead to accumulation of acetylcholine (ACh) in the cholinergic synapse and this in turn is likely to increase the activity of the acetylcholinesterase and thereby it might affect the neuronal transmission which may in turn affect the memory processes. Therefore, to validate this, we assessed the AChE activity in the prefrontal cortex and the hippocampus. Since CDRI-08 is in use for boosting the memory in normal human subjects, we also examined its direct effects on the normal control mice in addition to above.

Our radial arm maze (RAM) data from the acquisition experiment suggest that mice were able to learn well during the training period (Figure 1) and scopolamine treatment leads to decline in the learning for reference, working, and reference-working memory. Also, it suggests that mice treated with scopolamine may develop impairments in both working and reference memory by affecting the prefrontal cortex, which may later on form defective long-term memory in the hippocampus. This could be attributed to defective synaptic plasticity due to inactivation of acetylcholine receptor activity and/or altered NMDA receptors on the postsynaptic density in prefrontal cortex or the hippocampus. Mode of scopolamine action is known to block the muscarinic receptors acetylcholine receptors [42, 43] which ultimately lead to profound deficits in attention and memory by inhibiting cholinergic neurotransmission. Our data are consistent with the scopolamine-induced deficiency in the spatial memory [29]. The other possibility of memory deficit may be due to scopolamine-induced oxidative stress in the brain [44]. Available evidence also suggests that the memory deficiency might be due to effects of scopolamine in blocking NMDA receptors [34]. However, our data do not directly support this function of the scopolamine but it indicates their association.

Acetylcholine level is under the dynamic regulation of an enzyme AChE at the synapse [45]. Numerous studies have implicated the importance of acetylcholine in higher brain functions like learning and memory [46–48]. Alterations in the acetylcholine metabolism are also involved in various neuropathological conditions like mild cognitive impairment (MCI), Alzheimer's disease (AD), and dementia [49, 50]. Decreased levels of acetylcholine at the synaptic cleft have been implicated in the loss of synaptic architecture leading to state of amnesia in rodents [51]. In our study, activity of AChE was found to be elevated in scopolamine-treated group which suggests a rapid breakdown of the acetylcholine which might lead to decline in its level in the synapse which may further be correlated with decline in various memory types. The CDRI-08 treatment was found to reverse the level of AChE which further was correlated with recovery of memory close to that in the normal control mice. The CDRI-08 alone also leads to a significant decline in the AChE activity. This suggests that CDRI-08 effect on memory improvement is via its action on the AChE. Our study confirms the neuroprotective role of CDRI-08 in recovering the memory loss due to amnesia induced by scopolamine treatment. The precise mechanism of CDRI-08 effects on the regulation of AChE and memory formation as well as memory recovery process, however, is yet to be understood. Nonetheless, recent study on schizophrenic human subjects supports our finding on the modulation of cholinergic neuronal activities by CDRI-08 [32]. Over the past several decades, plethora of natural products like coumarins, flavonoids, and stilbenes have been in use for the treatment of cognitive dysfunctions by inhibiting AChE [52–54]. Our findings on the recovery of memory loss by* Bacopa monnieri* extract CDRI-08 also suggest that the CDRI-08 protects the memory loss or improves the cognitive functions of the brain abrogated by scopolamine by inhibiting the AChE activity in the prefrontal cortex and hippocampus. The CDRI-08 effect may also be brought by altering the choline acetyltransferase (CAT) activity and thereby the level of acetylcholine in the synapse. Our study requires to be further supplemented with a close examination of alterations in the CAT activity due to CDRI-08 which may provide an insight into its mechanism of action on the improvement of memory in either normal or amnesic subjects.

NMDA receptors are widely concentrated in the cortical region of brain and in hippocampus. Recent studies have correlated NMDA receptor GluN2B subunit with varieties of learning and memory functions in the hippocampus-dependent spatial memory [9, 55]. Loss of NMDA receptors (NMDARs) has been implicated in long-term depression (LTD), loss of synaptic plasticity, learning, memory, and progression of various neuropathological conditions [56, 57]. In our study, we observed that scopolamine-induced forgetfulness in mice, as evidenced by our study on behavioral analysis by radial arm maze test, is correlated with decline in expression of the GluN2B subunit in the prefrontal cortex as well as hippocampus. This may further be associated with decline in cholinergic function along with alterations in the GluN2B expression resulting into loss of memory reflected by significant increase in the reference, working, and reference-working memory errors by amnesic mice while performing on the radial arm maze. Further, treatment of scopolamine-induced amnesic mice with CDRI-08 upregulates the expression of GluN2B in both the brain structures which is associated with enhancement of various memory types. Also, the CDRI-08 alone has ability to elevate the expression of GluN2B subunit compared to the normal control mice. In a recent study, Krishnakumar et al. also demonstrated a significant decline in the expression of GluN2B in the cerebral cortex of pilocarpine-induced epileptic rats which was reversed by the treatment of* Bacopa monnieri* extract [58]. Our data do suggest that CDRI-08 is highly effective in improving and recovering the memory loss/deficiency which might be by positively regulating the synaptic plasticity, which would have been otherwise damaged by scopolamine leading to memory loss. Our expression study data, which corresponds to behavioral data on the performance of mice for various memory types, clearly suggests the use of CDRI-08 with its possible mechanism of action in restoration of memory loss. However,* Bacopa monnieri* extract has been shown to reverse the memory loss by decreasing the density of NMDAR in the prefrontal cortex and CA1 neuronal region of the hippocampus in phencyclidine-induced cognitive deficit rat model where its density was elevated during memory impairment [59]. Thus it is evident that CDRI-08 induced mechanisms for the recovery of memory loss (amnesia) involves alterations in the level of NMDAR. Role of CDRI-08 during recovery of memory loss due to scopolamine-induced amnesia by regulating the other neuronal systems such as GABAergic system [60] and involvement of various glutamate transporters and their regulation by CDRI-08 may also be importantly involved [59].

Based on the behavioral and molecular analysis, our study suggests that scopolamine-induced amnesia in mice is mediated via increase in the acetylcholinesterase activity and decrease in the population of NMDA type glutamate receptor in the glutamatergic synapse. Also, our study provides a molecular basis of the possible therapeutic action of a special* Bacopa monnieri* extract, CDRI-08, in the recovery of the scopolamine-induced memory deficit and its role in enhancing the levels of learning and memory in mice. However, to ascertain the precise role of NMDA receptor types and AChE in CDRI-08-mediated modulation of synaptic plasticity, neuronal cell density, dendritic arborization, dendritic spine density and their morphological aspects, and so forth is required to be thoroughly addressed. A thorough examination of the expression of other subunits of NMDAR such as GluN1, GluN2A, their trafficking [61], and assay of ChAT activity will be required to ascertain the mechanisms of action of the bacosides A and B rich CDRI-08 on the glutamatergic and cholinergic system, respectively, during its action on improvement of memory.

## Figures and Tables

**Table 1:** Schedule for the vehicle/drug treatment and training on Radial Arm Maze paradigm.

Experimental Set	Group (*N* = 7)	Treatment Schedule		
		Day 1–7	Day 8–15	Day 16–21
Control	I	0.5% Tween 80 (150 min before) + NS (120 min before)		

*B. monnieri* extract (CDRI-08)	II	0.5% Tween 80 (150 min before) + NS (120 min before)	*B. monnieri* extract (200 mg/Kg) in 0.5% Tween 80 (150 min before) + NS (120 min before)	

Scopolamine	III	0.5% Tween 80 (150 min before) + NS (120 min before)		Scopolamine (2 mg/Kg) in NS (120 min before) + 0.5% Tween 80 (150 min before)

Scopolamine + *B. monnieri *extract (CDRI-08)	IV	0.5% Tween 80 (150 min before) + Scopolamine (2 mg/Kg) in NS (120 min before)	*B. monnieri* extract (200 mg/Kg) in 0.5% Tween 80 (150 min before) + NS (120 min before)	

Note: NS-Normal saline; before-duration of time before behavioral recording.

## References

[1] Gazzaniga M. S., Ivry R. B., Mangun G. R. (2009). *Cognitive Neuroscience: The Biology of the Mind*.

[2] Lerner K. L., Lerner B. W. (2004). *Gale Encyclopedia of Science*.

[3] Goodwin D. W., Crane J. B., Guze S. B. (1969). Alcoholic ‘blackouts’: a review and clinical study of 100 alcoholics. *The American Journal of Psychiatry*.

[4] Köhr G. (2006). NMDA receptor function: subunit composition versus spatial distribution. *Cell and Tissue Research*.

[5] Tsien J. Z., Huerta P. T., Tonegawa S. (1996). The essential role of hippocampal CA1 NMDA receptor-dependent synaptic plasticity in spatial memory. *Cell*.

[6] Zoladz P. R., Park C. R., Halonen J. D. (2012). Differential expression of molecular markers of synaptic plasticity in the hippocampus, prefrontal cortex, and amygdala in response to spatial learning, predator exposure, and stress-induced amnesia. *Hippocampus*.

[7] Cull-Candy S., Brickley S., Farrant M. (2001). NMDA receptor subunits: diversity, development and disease. *Current Opinion in Neurobiology*.

[8] Gielen M., Retchless B. S., Mony L., Johnson J. W., Paoletti P. (2009). Mechanism of differential control of NMDA receptor activity by NR2 subunits. *Nature*.

[9] Wyllie D. J. A., Livesey M. R., Hardingham G. E. (2013). Influence of GluN2 subunit identity on NMDA receptor function. *Neuropharmacology*.

[10] Zhang X.-H., Liu S.-S., Yi F., Zhuo M., Li B.-M. (2013). Delay-dependent impairment of spatial working memory with inhibition of NR2B-containing NMDA receptors in hippocampal CA1 region of rats. *Molecular Brain*.

[11] Ling W., Chang L., Song Y. (2012). Immunolocalization of NR1, NR2A, and PSD-95 in rat hippocampal subregions during postnatal development. *Acta Histochemica*.

[12] Sepulveda F. J., Bustos F. J., Inostroza E. (2010). Differential roles of NMDA receptor subtypes NR2A and NR2B in dendritic branch development and requirement of RasGRF1. *Journal of Neurophysiology*.

[13] Gambrill A. C., Barria A. (2011). NMDA receptor subunit composition controls synaptogenesis and synapse stabilization. *Proceedings of the National Academy of Sciences of the United States of America*.

[14] Jo Y. S., Choi J. S. (2014). Memory retrieval in response to partial cues requires NMDA receptor-dependent neurotransmission in the medial prefrontal cortex. *Neurobiology of Learning and Memory*.

[15] Fan M. M. Y., Fernandes H. B., Zhang L. Y. J., Hayden M. R., Raymond L. A. (2007). Altered NMDA receptor trafficking in a yeast artificial chromosome transgenic mouse model of Huntington's disease. *Journal of Neuroscience*.

[16] Huo X.-L., Min J.-J., Pan C.-Y. (2014). Efficacy of lovastatin on learning and memory deficits caused by chronic intermittent hypoxia-hypercapnia: through regulation of NR2B-containing NMDA receptor-ERK pathway. *PLoS ONE*.

[17] Bagetta V., Ghiglieri V., Sgobio C., Calabresi P., Picconi B. (2010). Synaptic dysfunction in Parkinson's disease. *Biochemical Society Transactions*.

[18] Picconi B., Piccoli G., Calabresi P. (2012). Synaptic dysfunction in Parkinson’s disease. *Synaptic Plasticity*.

[19] Brazell C., Preston G. C., Ward C., Lines C. R., Traub M. (1989). The scopolamine model of dementia: chronic transdermal administration. *Journal of Psychopharmacology*.

[20] Ebert U., Kirch W. (1998). Scopolamine model of dementia: electroencephalogram findings and cognitive performance. *European Journal of Clinical Investigation*.

[21] Aguiar S., Borowski T. (2013). Neuropharmacological review of the nootropic herb *Bacopa monnieri*. *Rejuvenation Research*.

[22] Shinomol G. K., Muralidhara, Bharath M. M. S. (2011). Exploring the role of ‘Brahmi’ (*Bocopa monnieri* and *Centella asiatica*) in brain function and therapy. *Recent Patents on Endocrine, Metabolic and Immune Drug Discovery*.

[23] Deepak M., Amit A. (2004). The need for establishing identities of ‘bacoside A and B’, the putative major bioactive saponins of Indian medicinal plant *Bacopa monnieri*. *Phytomedicine*.

[24] Sivaramakrishna C., Rao C. V., Trimurtulu G., Vanisree M., Subbaraju G. V. (2005). Triterpenoid glycosides from *Bacopa monnieri*. *Phytochemistry*.

[25] Murthy P. B. S., Raju V. R., Ramakrisana T. (2006). Estimation of twelve bacopa saponins in *Bacopa monnieri* extracts and formulations by high-performance liquid chromatography. *Chemical and Pharmaceutical Bulletin*.

[26] Jyoti A., Sethi P., Sharma D. (2007). Bacopa monniera prevents from aluminium neurotoxicity in the cerebral cortex of rat brain. *Journal of Ethnopharmacology*.

[27] Shinomol G. K., Mythri R. B., Srinivas Bharath M. M. (2012). Bacopa monnieri extract offsets rotenone-induced cytotoxicity in dopaminergic cells and oxidative impairments in mice brain. *Cellular and Molecular Neurobiology*.

[28] Le X. T., Pham H. T. N., Do P. T. (2013). *Bacopa monnieri* ameliorates memory deficits in olfactory bulbectomized mice: possible involvement of glutamatergic and cholinergic systems. *Neurochemical Research*.

[29] Saraf M. K., Prabhakar S., Khanduja K. L., Anand A. (2011). *Bacopa monniera* attenuates scopolamine-induced impairment of spatial memory in mice. *Evidence-Based Complementary and Alternative Medicine*.

[30] Liu X., Yue R., Zhang J., Shan L., Wang R., Zhang W. (2013). Neuroprotective effects of bacopaside i in ischemic brain injury. *Restorative Neurology and Neuroscience*.

[31] Pase M. P., Kean J., Sarris J., Neale C., Scholey A. B., Stough C. (2012). The cognitive-enhancing effects of bacopa monnieri: a systematic review of randomized, controlled human clinical trials. *Journal of Alternative and Complementary Medicine*.

[32] Peth-Nui T., Wattanathorn J., Muchimapura S. (2012). Effects of 12-week *Bacopa monnieri* consumption on attention, cognitive processing, working memory, and functions of both cholinergic and monoaminergic systems in healthy elderly volunteers. *Evidence-Based Complementary and Alternative Medicine*.

[33] Saraf M. K., Anand A., Prabhakar S. (2010). Scopolamine induced amnesia is reversed by *Bacopa monniera* through participation of kinase-CREB pathway. *Neurochemical Research*.

[34] Falsafi S. K., Deli A., Höger H., Pollak A., Lubec G. (2012). Scopolamine administration modulates muscarinic, nicotinic and nmda receptor systems. *PLoS ONE*.

[35] Buresova O., Bures J. (1981). Role of olfactory cues in the radial maze performance of rats. *Behavioural Brain Research*.

[36] Mizumori S. J. Y., Channon V., Rosenzweig M. R., Bennett E. L. (1987). Short- and long-term components of working memory in the rat. *Behavioral Neuroscience*.

[37] Olton D. S. (1987). The radial arm maze as a tool in behavioral pharmacology. *Physiology and Behavior*.

[38] Srikumar B. N., Ramkumar K., Raju T. R., Shankaranarayana Rao B. S. (2004). Assay of acetylcholinesterase activity in the brain. *Brain and Behavior*.

[39] Ellman G. L., Courtney K. D., Andres V., Featherstone R. M. (1961). A new and rapid colorimetric determination of acetylcholinesterase activity. *Biochemical Pharmacology*.

[40] Bradford M. M. (1976). A rapid and sensitive method for the quantitation of microgram quantities of protein utilizing the principle of protein dye binding. *Analytical Biochemistry*.

[41] Singh K., Gaur P., Prasad S. (2007). Fragile x mental retardation (Fmr-1) gene expression is down regulated in brain of mice during aging. *Molecular Biology Reports*.

[42] Wallace T. L., Bertrand D. (2013). Importance of the nicotinic acetylcholine receptor system in the prefrontal cortex. *Biochemical Pharmacology*.

[43] Vamvakidès A. (2003). Selective M1 muscarinic agonists: failure of therapeutic strategy against Alzheimer's disease or inappropriate tactics?. *Annales Pharmaceutiques Francaises*.

[44] Giridharan V. V., Thandavarayan R. A., Sato S., Ko K. M., Konishi T. (2011). Prevention of scopolamine-induced memory deficits by schisandrin B, an antioxidant lignan from *Schisandra chinensis* in mice. *Free Radical Research*.

[45] Picciotto M. R., Higley M. J., Mineur Y. S. (2012). Acetylcholine as a neuromodulator: cholinergic signaling shapes nervous system function and behavior. *Neuron*.

[46] Zhou X., Qi X. L., Douglas K. (2011). Cholinergic modulation of working memory activity in primate prefrontal cortex. *Journal of Neurophysiology*.

[47] Gold P. E. (2003). Acetylcholine modulation of neural systems involved in learning and memory. *Neurobiology of Learning and Memory*.

[48] Easton A., Douchamps V., Eacott M., Lever C. (2012). A specific role for septohippocampal acetylcholine in memory?. *Neuropsychologia*.

[49] Muir J. L. (1997). Acetylcholine, aging, and Alzheimer's disease. *Pharmacology Biochemistry and Behavior*.

[50] Ogawa N. (1989). Central acetylcholinergic systems in the normal aged and in the patient with Alzheimer-type dementia (ATD). *Rinsho Shinkeigaku*.

[51] Pepeu G., Giovannini M. G. (2004). Changes in acetylcholine extracellular levels during cognitive processes. *Learning & Memory*.

[52] Kar A., Panda S., Bharti S. (2002). Relative efficacy of three medicinal plant extracts in the alteration of thyroid hormone concentrations in male mice. *Journal of Ethnopharmacology*.

[53] Wang X., Wang L. P., Tang H. (2014). Acetyl-l-carnitine rescues scopolamine-induced memory deficits by restoring insulin-like growth factor II via decreasing p53 oxidation. *Neuropharmacology*.

[54] Jahanshahi M., Nickmahzar E. G., Babakordi F. (2013). The effect of *Ginkgo biloba* extract on scopolamine-induced apoptosis in the hippocampus of rats. *Anatomical Science International*.

[55] Plattner F., Hernández A., Kistler T. M. (2014). Memory enhancement by targeting Cdk5 regulation of NR2B. *Neuron*.

[56] Brim B. L., Haskell R., Awedikian R. (2013). Memory in aged mice is rescued by enhanced expression of the GluN2B subunit of the NMDA receptor. *Behavioural Brain Research*.

[57] Kuehl-Kovarik M. C., Magnusson K. R., Premkumar L. S., Partin K. M. (2000). Electrophysiological analysis of NMDA receptor subunit changes in the aging mouse cortex. *Mechanisms of Ageing and Development*.

[58] Krishnakumar A., Anju T. R., Abraham P. M., Paulose C. S. (2015). Alteration in 5-HT2C, NMDA receptor and IP3 in cerebral cortex of epileptic rats: restorative role of *Bacopa monnieri*. *Neurochemical Research*.

[59] Piyabhan P., Wetchateng T., Sirseeratawong S. (2013). Cognitive enhancement effects of *Bacopa monnieri*(Brahmi) on novel object recognition and NMDA receptor immunodensity in the prefrontal cortex and hippocampus of sub-chronic phencyclidine rat model of schizophrenia. *Journal of the Medical Association of Thailand*.

[60] Mathew J., Balakrishnan S., Antony S., Abraham P., Paulose C. S. (2012). Decreased GABA receptor in the cerebral cortex of epileptic rats: effect of *Bacopa monnieri* and Bacoside-A. *Journal of Biomedical Science*.

[61] Pandey S. P., Rai R., Gaur P., Prasad S. (2015). Development- and age-related alterations in the expression of AMPA receptor subunit GluR2 and its trafficking proteins in the hippocampus of male mouse brain. *Biogerontology*.

